# Risks of emergency use authorizations for medical products during outbreak situations: a COVID-19 case study

**DOI:** 10.1186/s12938-020-00820-0

**Published:** 2020-10-02

**Authors:** Almir Badnjević, Lejla Gurbeta Pokvić, Zijad Džemić, Fahir Bečić

**Affiliations:** 1 Medical Device Inspection Laboratory Verlab, Sarajevo, Bosnia and Herzegovina; 2 Faculty of Pharmacy Sarajevo, University of Sarajevo, Sarajevo, Bosnia and Herzegovina; 3 International Burch University, Sarajevo, Bosnia and Herzegovina; 4 Institute of Metrology of Bosnia and Herzegovina, Sarajevo, Bosnia and Herzegovina

**Keywords:** Medical device, Outbreak, COVID-19, Regulatory framework, Import, Market placement

## Abstract

**Background:**

The world is facing an unprecedented outbreak affecting all aspects of human lives which is caused by the COVID-19 pandemic. Due to the virus novelty, healthcare systems are challenged by a high rate of patients and the shortage of medical products. To address an increased need for essential medical products, national authorities, worldwide, made various legislative concessions. This has led to essential medical products being produced by automotive, textile and other companies from various industries and approved under the emergency use authorizations or legal concessions of national regulatory bodies. This paper presents a narrative commentary of the available documentation on emergency use authorizations and legal concessions for medical products during COVID-19 pandemic.

**Methodology:**

The basis for narrative commentary includes scientific articles published in Web of Science, Scopus, PubMed and Embase databases, official publications of international organizations: Food and Drug Agency (FDA), World Health Organisation (WHO), World Bank and United Nations (UN), and national regulatory agency reports in native languages (English, German, Bosnian, and Croatian) published from November 1, 2019 to May 1, 2020. This paper focuses on three types of essential medical products: mechanical ventilators, personal protective equipment (PPE) and diagnostic tests. Evidence-informed commentary of available data and potential identified risks of emergency use authorizations and legal concessions is presented.

**Discussion:**

It is recognized that now more than ever, raising global awareness and knowledge about the importance of respecting the essential requirements is needed to guarantee the appropriate quality, performance and safety of medical products, especially during outbreak situation, such as the COVID-19 pandemic. Emergency use authorizations for production, import and approval of medical products should be strictly specified and clearly targeted from case to case and should not be general or universal for all medical products, because all of them are associated with different risk level.

**Conclusion:**

Presented considerations and experiences should be taken as a guide for all possible future outbreak situations to prevent improvised reactions of national regulatory bodies.

## Background

Severe Acute Respiratory Syndrome Coronavirus 2 (SARS-CoV-2) caused thousands of people in need of hospitalizations in healthcare institutions simultaneously [1]. As soon as the problem with SARS-CoV-2-associated disease known as COVID-19 emerged, recommendations and guidelines for patient treatments and essential medical products were published by various bodies such as the World Health Organisation (WHO) [2], the United Kingdom National Institute for Health and Care Excellence (NICE) [3], the United States Food and Drug Administration (FDA) [4], and the National Health Commission of the People’s Republic of China [5]. These guidelines had changed daily to tackle this disease in most effective ways.

In the early days of the pandemic, many COVID-19 guidelines implied a mandatory use of mechanical ventilators for the treatment of patients with severe symptoms. These recommendations also implied the use of personal protective equipment (PPE) in the community, home care and in health care settings. Mechanical ventilators, personal protective equipment (PPE) and diagnostic tests had soon become essential medical products in COVID-19 response [6].

These guidelines and recommendations led to the unprecedented and almost immediate shortage of these medical products with huge socio-economic implications worldwide [7]. Since the medical product industry was not able to answer this growing need, many world countries restricted or even banned the export of medical products, causing the countries without their own medical production industry to face an extremely huge challenge. So, to answer this healthcare need and associated social fears, many industries such as automotive, textile and military stopped their primary activities and adapted their production.

For example, in Spain, automotive manufacturer Seat adjusted their assembly lines in Martorell facilities for emergency ventilator production [8]. Spanish Agency of Medicines and Medical Devices released these devices for clinical investigations. In Italy, the army joined the industry to help in various ventilator production lines to foster the need for these essential devices [9]. In the USA, textile giant in Middlesex produced face masks to be used by medical professionals fighting COVID-19 [10]. One of the leading manufacturers of medical products, Medtronic, made available the full design specifications, production manuals, design documents and, software code for one of their models of portable ventilators [11]. Also, many companies shared the news about developed prototypes available for the use in the healthcare institutions. Tech company Nvidia responded to the crisis by releasing an open-source ventilator hardware design which can be assembled quickly, using off-the-shelf parts [12]. Many other open-sourced mechanical ventilator designs became available too, as it is elaborated in the study by Pearce [13]. In many countries such as South Korea, companies started the production of COVID-19 tests for screening, monitoring and diagnosis [14]. To avoid false test results, WHO published various guidelines for laboratories providing various tests for COVID-19 [2].

At the end, this resulted in chaotic situation, especially in countries without its own medical product industry. As a consequence of this, essential medical products of questionable quality and safety, approved by emergency use authorizations or legal concessions, are available on the market and in the healthcare institutions. For instance, in the United Kingdom, doctors warned that some emergency-approved ventilators could kill patients if used in hospitals [15] and in Bosnia and Herzegovina, there were ventilators stopped at the national border due to complications with emergency use authorizations issued by national regulatory authorities [16].

Globally, the increased need for healthcare revealed the same systematic weaknesses in terms of system response. Apart from the lack of medical professionals available to manage high number of patients, which was, from this point of view, reasonably expectable, response to shortage of essential medical products [17, 18] was the major weakness of healthcare system COVID-19 response, which could have been avoided if rules, guidelines and procedures for emergency production, approval and management of essential medical products existed. COVID-19 case showed that raising the global awareness and knowledge about the importance of respecting the essential requirements is a precondition for assuring the appropriate quality, performance and safety of medical products, especially in case of high demand such as this COVID-19 outbreak [7].

This paper gives an evidence-informed narrative commentary of available references data and information available and potential identified risks on emergency use authorizations and legal concessions for medical products during COVID-19 pandemic.

## Methodology

### Data

This narrative commentary is based on the data that were previously published in the form of:

- scientific articles published in Web of Science, Scopus, PubMed and Embase databases,
- official publications of international organizations: Food and Drug Agency (FDA), World Health Organization (WHO), World Bank and United Nations (UN),
- National regulatory agency reports (emergency use authorizations and legal concessions) in native languages (English, German, Bosnian, and Croatian).

Data published from November 1, 2019 to May 1, 2020 were taken into consideration. The data referenced in this narrative commentary includes also information presented in published news during stated timeframe.

The commentary was done on three types of essential medical products: (1) mechanical ventilators, (2) personal protective equipment (PPE) and (3) diagnostic tests.

The databases of scientific articles were searched by following keywords: *emergency use authorization, emergency approvals, COVID*-*19, declaration of conformity, diagnostic tests, PPE and mechanical ventilators, medical device approval*. Exclusion criteria were related to the language of material.

Inclusion criteria for scientific articles were that they were published in English, German, Croatian and Bosnian language in the above-mentioned timeframe. Additionally, authors manually screened reference lists of related published materials for additional relevant studies.

The data gathering was conducted independently by two authors, while quality assessment of the materials was done jointly by two authors. Two authors (AB and LGP) independently identified potential studies by reading the title and abstract, and any disagreement was settled through the discussion with the third and fourth authors (ZDz and FB).

### Risk analysis methodology

To analyze the potential risks of emergency use authorizations and legal concessions published during COVID-19 essential medical product shortage period, the first outline is the standardized process for two major jurisdictions (European Union and United States of America). Afterwards, a review on how they have been alternated or adapted in light of COVID-19 pandemic is presented.

To asses a risk level, based on probability and consequence severity, the risk matrix [19] was used, as shown in Table 1. The risk priority number (RPN) is calculated as the probability multiplied by severity, and according to this number, risks can be classified as low, medium and high as shown in Table 2.

**Table 1:** Risk assessment matrix: severity vs. probability [19]

	Probability				
	Criteria	1 Rarely	2 Not often	3 Often	4 Certainly
Severity	4 Critical	4	8	12	16
	3 Significant	6	6	9	12
	2 Small	4	4	6	8
	1 None	2	2	3	4

**Table 2:** Risk level classification [19]

Risk priority number (RPN)	Risk category	Decision on admissibility of risk
1–4	Low	The risk is acceptable. No further action is required
5–9	Medium	Risk mitigation measures should be investigated to determine if the risk can be reduced. If the risk cannot be reduced, the risk can be accepted and the decision to accept must be properly documented
10–16	High	Risk mitigation procedures are required. If the high risk cannot be reduced, formal acceptance and documentation of the risk by appropriate decision makers (including members of the quality department) is required

In terms of essential medical products, actions categorized with a high level of risk are very likely to cause death or serious injury/illness.

Below, risk analysis of identified concessions and emergency use authorization is presented separately for mechanical ventilators, PPE and COVID-19 tests.

## Results and discussion

The narrative commentary presented below takes into account scientific articles [13, 17, 20–23], official publications of Food and Drug Agency (FDA) [4, 24], European Commission [25], United Nations [26] and national regulatory agency reports in native languages [27, 28].

### International regulation procedures for medical products

A regulatory framework for medical products is a very complex system, but designed to ensure the safety of all involved stakeholders, from manufacturers, healthcare professionals to patients. Medical products in the European Union are regulated by Medical Device Directives [19] and international guidelines [29, 30]. From 2021., new Medical Device Regulation (MDR) [31, 32], which is much more strict than previous Directives, will be enforced. Within legislation requirements, all aspects of the medical product life-cycle are defined. The compliance with the above-mentioned legislation is assured by the CE marking. The CE mark is obtained after a product assessment is performed by a designated notified body [33]. A notified body issues Certificate of Conformity (CoC), stating its opinion about the limited aspects of conformity. Currently, in the EU, there are 12 designated notified bodies for 90/385/EEC Directive, 55 for 93/42/EEC and only 13 for Medical Device Regulation (EU) 2017/745 [34].

The Regulation itself refers to harmonized standards, as a way in which manufacturers or notified bodies can assess the conformity of a product. The pathway that manufacturers can take for achieving the CE marking is different for COVID-19 essential medical products depending on their classification as shown in the Table 3.

**Table 3:** Categorization of COVID-19 essential medical products according to EU and FDA legislation [35]

Type of medical product	EU classification	FDA classification	Description
Mechanical ventilators	MD Class IIb	MD Class II	Medium- to high-risk devices, and patients may use them for a period longer than 30 days
Personal protective equipment (PPE)	MD Class I	MD Class I	Low or moderate risk to patient’s health and safety not intended for use in supporting or sustaining life or of substantial importance in preventing impairment to human heal
Diagnostic tests	IVD Class I	IVD Class I	No public health risk or low personal risk

During protection against the COVID-19, the PPEs are classified as medical products with the highest risk class (class II or III). Product compliance with the harmonised standards requirements is demonstrated not only by manufacturers, but also by authorized notified bodies.

However, the CoC is not the only document needed to guarantee the product conformity to legislation and standards. It works along with a Declaration of Conformity (DoC). According to the European legislation, this is a formal declaration issued by a manufacturer, or the manufacturer’s representative, that the product to which it applies meets all the relevant requirements defined by the legislation and standards. This document is a sole responsibility of the manufacturer in which the manufacturer assumes responsibility for the compliance of the product. This document is maintained with the technical documentation for the product and should be presented to national authorities during the import, and per request in other cases. The final step is complying with the national requirements which refer to completing an additional registration with National Competent Authorities by manufacturer or its legal representative. This is the overall procedure on how conformity of a product, especially medical products, is ensured in normal situations [30–33, 36]. In ordinary conditions, a typical timeframe for the Notified/Certification Body is 6–9 months, as the preparatory work for certification of medical product and its market placement takes more than 6 months [34].

Similarly, in the United States, the FDA approval process can take between one week and eight months, but the overall process of bringing a medical device to the market, from a concept to approval, takes from 3 to 7 years [4]. By the FDA regulation, domestic and foreign manufacturers, as well as initial distributors and importers of medical devices, must register their establishments with the FDA. Afterwards, manufacturers must list devices they intend to sell with the FDA because medical device cannot be commercially distributed in the USA market until Premarket Notification 510(k) is obtained. Cases of post-market surveillance incidents, in which a device may have caused or contributed to a death or a serious injury, are being reported to FDA under the Medical Device Reporting program.

### Inadequate emergency use authorizations for approval and import of medical products during COVID-19 pandemic

Most countries where national bodies, such as the Civil Protection and/or noncompetent Crisis Headquarters took a national lead during the COVID-19 pandemic, have caused inadequate procurements, approvals and import of non-compliant essential medical products. Some of the risk examples identified in the previous period are presented in the Table below.

#### Mechanical ventilator

Mechanical ventilators are categorized as Class II devices (Table 3), the usage of which in clinical settings poses medium to high risk to patients. Due to this inherited risk, prior to a market placement, mechanical ventilators are subjected to rigorous and lengthy clinical trials and regulatory processes. The outcome of these processes is clear technical notes and proofs of clinical effectiveness that are all part of authorizations for their usage. In urgent cases, these lengthy procedures can be shortened. For instance, in the USA, FDA’s 510(k) policy [4] allows manufacturers to place devices, including mechanical ventilators, on the market after a shorter testing. However, clinical evaluations must be conducted, but they are aimed at showing that a new device is sufficiently similar to a product that is already on the market. This principle is known as “substantial equivalence” [35]. There is a similar case in the European regulation [31, 32] and it is known as “demonstration of equivalence”. These authorizations refer to new products with only small feature modifications in respect to the already accepted model. This approach is very useful in a crisis situation, when manufacturers need to speed up the production to foster the need for medical products on the market.

During the COVID-19 pandemic, leading regulatory authorities from developed, high-income countries have taken a series of measures to tackle the need of the market for essential medical products, including mechanical ventilator shortage.

In the USA, FDA issued emergency use authorizations for essential medical products to be used during the COVID-19 crisis. These regulations for mechanical ventilators [35] gave instructions on how to overcome the shortage by modifying intended usage of already approved devices, such as anaesthesia gas machines and continuous positive airway pressure breathing devices to complement the purpose of mechanical ventilators. Also, emergency use authorizations allowed manufacturer to make small modifications on already approved products during production. For instance, a change of materials and parts. FDA defines a validation process for manufacturers and a basic safety, performance and labelling criteria. FDA defined a clear framework on importing new devices in the US market.

In the EU, the European Commission announced a recommendation for its member states. According to their recommendations, some medical devices are to be exempted from normal procedures and could be placed on the market without previous CE certification [37]. However, defining details of those recommendations has been left to each state’s competent authority. These approaches have found to be very successful, because national regulatory authorities had not only well defined all procedures, minimum technical specifications and safety considerations to guide manufacturers during this crisis, but have also maintained satisfying level of compliance of new devices. Applying the stated measures, governments had taken on some of the operational risks and the manufacturers of medical devices are still legally responsible for any patient harm because of the device design.

However, in some middle- and low-income countries, the lack of this strategic approach and competent national regulatory authority has led to various concessions of ordinary medical product-related procedures, including mechanical ventilators during the COVID-19 crisis. Table 4 presents the identification of measures that were made by few crisis headquarters which led to decreasing quality of medical products available on the market.

**Table 4:** Risk assessment of emergency use authorizations for mechanical ventilators during the COVID-19 crisis

No.	Emergency use authorization measure	Consequence	Severity (1–4)	Probability (1–4)	Risk level (low, medium, high)
1	Import without CE mark and/or Declaration of conformity [38]	Unknown manufacturer Unknown applied standards and directives Missing test results Responsible person	4	3	High
2	Product without national manufacturer representative [16]	Missing installation of the product Missing education about the product Missing urgent service Missing preventive service inability to use the product non-functional usage of the product	4	3	High
3	Fast approval of prototype	Legal and health consequences	4	4	High
4	Change of prescribed usage [39]	Lowering the capacity of healthcare system usage out of intended scope	2	4	Medium
5	Modification of existing functions by manufacturer [40]	Lower quality of the product restricted functionality	1	4	Low

#### Personal protective equipment (PPE)

PPE is classified as Class I medical product (Table 1), the usage of which in clinical settings ensures the safety of medical professionals during any patient treatment. These medical products carry low risk when used by its users. Although the risk is low, it cannot be neglected, and a number of legal requirements are defined. These legal requirements are published as policies, directives or regulations.

Similar to mechanical ventilators, as soon as shortage of PPE was announced during the COVID-19 pandemic, many companies started with production and sales of PPEs. To maintain the quality assurance of products, leading regulatory bodies, such as EC and FDA published emergency use authorizations and Quality Systems Regulations and Good Manufacturing Practices to help ensure that the newly produced PPE is safe and effective, and complies with minimum requirements for safety and performance.

However, in some middle- and low-income countries, national regulatory authorities and crisis headquarters have not published any recommendations and had made several concessions from ordinary procedures, allowing PPE of questionable quality and effectiveness to enter the market, either by being sold to users or being offered as donation to healthcare institutions. Some of the cases recorded during the COVID-19 pandemic and their risk assessment are presented in the Table 5.

**Table 5:** Risk assessment of emergency use authorizations for PPE during the COVID-19 crisis

No.	Emergency use authorization measure	Consequence	Severity (1–4)	Probability (1–4)	Risk level (low, medium, high)
1	Import without CE mark and/or Declaration of conformity [41, 42]	Unknown manufacturer Unknown applied standards and directives Missing test results Responsible person	3	3	Medium
2	Product without national manufacturer representative	Longer procurement procedures	3	3	Medium
3	Fast approval of product	legal and health consequences	1	4	Low

#### Diagnostic tests

After first responses of healthcare systems to a growing number of patients during the COVID-19 pandemic, the focus shifted on finding diagnostic and screening tests. The first technique used to test for the presence of this virus was quantitative Reverse Transcription Polymerase Chain Reaction, or qtr.-PCR [43]. This kind of testing is very labour-intensive and may lead to false-positive or false-negative test results [44]. All leading bio-tech and pharmaceutical companies aimed their efforts at innovating devices and diagnostic tests. FDA and EC published recommendations for the COVID-19 tests requiring that for a test to be placed on the market, manufacturer needs to provide a technical file demonstrating that the test is safe and performs as intended. Table 6 presents the risk of emergency use authorizations for diagnostic tests during the COVID-19 crisis.

**Table 6:** Risk assessment of emergency use authorizations for diagnostic tests during the COVID-19 crisis

No.	Emergency use authorization measure	Consequence	Severity (1–4)	Probability (1–4)	Risk level (low, medium, high)
1	Import without CE mark and/or Declaration of Conformity [45]	Unknown manufacturer Unknown applied standards and directives Missing test results Responsible person	4	3	High
2	Fast approval of prototype	Legal and health consequences False-negative results due to the questionable accuracy	4	4	High
3	Product without national manufacturer representative [46]	Missing instructions for the product Missing the education about the product	3	3	Medium

The outbreak situation known as COVID-19 has shown clear deviations from the essential medical product’s regulatory framework. This is an everyday situation for professionals working in low resource settings, such as low- and middle-income countries, and it had now emerged powerfully for the high-income countries during the COVID-19 pandemic as well. The COVID-19 pandemic and low resource settings have deep differences, but many commonalities too. Low resource settings are not only common in low- and middle-income countries, but also exist in rural and remote areas of many high-income ones. Contrary to the common belief, the main problem of low-resource settings lies beyond the lack of funding, clinical knowledge, specialised clinical personnel and technical staff, scarcity of medical devices, drugs and spare parts due to a jeopardized supply chain. As argued above, COVID-19 has simultaneously hindered the supply chain, increased the ICU hospitalization demand and reduced staff. This created de facto conditions that are quite common in low-resource settings. Still, the valid European regulatory framework was written mainly to protect European manufacturers from the unsustainable competition from manufacturers producing abroad. This evolution has also been driven by the manufacturers’ need to produce essential medical products for the widest market possible, therefore following the principle of generalization as opposed to particularism. The prevalence of generalization over particularism resulted in a loss of universality, and in the fact that regulatory procedures that can be sustained in normal conditions, at least by high-income countries, became unsustainable in times of crisis. The COVID-19 pandemic demonstrated the lack of knowledge and lack of preparedness of many high-income countries, aggravated by the slowness in perceiving the complexity of the situation faced by other countries, affected by the COVID-19 months in advance.

As it can be seen from Tables 4, 5 and 6, essential medical products carry different associated risks when observed in ordinary situations; thus, any exemption action and emergency use authorizations impose different levels of risk in the overall system. In the situations when the world is facing with a novel virus, diagnostic and screening tests become of vital importance for assessing the impact of outbreak as well as to plan on measures to contain the outbreak. In those situations, medical products such as diagnostic tests that are normally classified as low-risk products, by the effect on the patient, become crucial. Any uncertainty of these tests can have devastating effects. For instance, false-negative test results contribute highly to the increase of infected patients [47]. For this reason, emergency use authorizations associated with this kind of essential medical products carry high risks, which means that a clear and narrow framework needs to be defined by the regulatory authority in order to mitigate such situations. One way of mitigation is immediate definition of a national reference laboratory to cooperate with the WHO and disseminate related information clearly and unambiguously through the command chain. Due to its sensitivity, a national authority body should set up an inventory of all public and private resources available for performing diagnostic tests under the same standardized procedures. In this way, the national authority body is joining available forces within private and public healthcare sectors and increasing the reliability of conducted tests, while increasing the rate of tested patients. Such measures could help in shortening the time of global lock-down and maintaining the world industry and, thus, economy.

As increased testing for COVID-19 has major effect on restoring the “normal” world activities, mechanical ventilators have major effect in maintaining the responsiveness of the healthcare sector to the most affected patients, the ones with severe symptoms. As it can be seen from the Table 1, in ordinary situations, these devices are classified as the ones with a high risk, meaning that their functionality has the major impact on patients’ reaction; so any emergency use authorizations should be carefully defined in the time of crisis to avoid the general opinion that “anyone can produce ventilators under any circumstances.” Generalizing such opinion crates a common disbelief that who is producing the ventilators is of more importance than assessing the real number available in private and public healthcare systems. This race usually ends in having ventilators without necessary approval [47] or imported ventilators of questionable quality. It should be emphasized that ventilators without conformity declaration, spare parts available and service back-up available delivered to healthcare institutions become just an inventory stock inappropriate for the patient use. The crisis showed the importance of a national medical device inventory database. Having this database with detailed information on device type, status and location within the country, optimized management of medical product shortage could have been done. Statistics of the COVID-19 outbreak show that there was a different rate in different parts of the world, so, this means, when lowered to the national level, this inventory database would be helpful in allocating the ventilators to places where they are really needed. Such database was available in Bosnia and Herzegovina [44], but due to the lack of knowledge from the National Crisis Headquarters, it was not used at all in this manner during the crisis.

In the end, having information about infected patients and having ventilators that treat the severe cases are irrelevant, if basic medical products with the lowest associated risk in ordinary situations are not available. PPE is what makes healthcare system work continuously at full capacity. PPE is crucial for maintaining health of medical workers that have the knowledge and ability to perform tests and use ventilators. Therefore, this analysis showed that, in countries which do not have the medical device industry, a national regulatory body should be focused on establishing a functional production plant of essential PPE, with all relevant compliances. Textile industry and few other industry plants have shown a great capability in converting to the PPE production, and since these products are Class I products, there is less risk in emergency use authorizations, when essential performance tests are defined [48], for locally produced PPE instead of ventilators which will be proclaimed non-compliant for healthcare usage once the pandemic is over. Basically, for any emergency-produced medical devices and/or “low-tech” solutions to have a real impact, it is essential for them to coalesce around approved designs.

The key limitation of this review article is that the risk assessment is based on the limited number of risk sources.

In this paper, we propose that all emergency use authorizations for import and approval of medical products should be narrowly specified and clearly targeted from case to case and should not be general or universal for all medical products, because all of them are carrying different risks. Based on the risk analysis (Tables 4, 5 and 6) conducted in this article our recommendations are:

1. To define and make available minimum technical specifications for essential medical products and treatments;
2. To define shorter versions of emergency use authorizations for essential medical products with procedures;
3. To define minimum requirements for production of essential medical products in extraordinary situation for non-medical manufacturers;
4. To create national/international inventory database of medical products.

This is a set of general recommendations that can be adopted by national regulatory bodies to overcome relevant future situations.

## Conclusions

In this manuscript, through the performed risk assessment analysis on the three examples of COVID-19 essential medical products, we presented the risk levels based on emergency use authorizations issued in this outbreak situation. Based on the risk analysis, it can be seen that the authorizations associated with PPE have the lowest level of risk, but have a great impact on the overall population and functionality of the healthcare system, along with authorizations regarding diagnostic tests.

For diagnostic tests, a standardized approach is crucial to foster a higher rate of reliable tests performed.

As for ventilators—the world-wide panic has been caused. Relevant experts were rarely asked by the national regulatory bodies to consult on defining technical requirements and action steps regarding either procurement or installation, which led to the waste of public funds on what are now shown to be unusable medical products.

The main lesson that our community should learn from the COVID-19 pandemic experience is that there is a major need for an evidence-based regulatory framework.

## Data Availability

Not applicable.

## Abbreviations

PPE: Personal protective equipment
WHO: World Health Organisation
NICE: United Kingdom National Institute for Health and Care Excellence
FDA: United States Food and Drug Administration
CoC: Certificate of Conformity
DoC: Declaration of Conformity
MD: Medical Device
MDR: Medical Device Regulation
RPN: Risk priority number
qRT-PCR: Reverse Transcription Polymerase Chain Reaction
